# Indirect association and ranking hypotheses for literature based discovery

**DOI:** 10.1186/s12859-019-2989-9

**Published:** 2019-08-15

**Authors:** Sam Henry, Bridget T. McInnes

**Affiliations:** Department of Computer Science, Virginia Commonwealth University, 601 W. Main St. Rm 435, Richmond, 23284 USA

**Keywords:** Literature based discovery, Indirect association, Semantic relatedness, Semantic similarity

## Abstract

**Background:**

Literature Based Discovery (LBD) produces more potential hypotheses than can be manually reviewed, making automatically ranking these hypotheses critical. In this paper, we introduce the indirect association measures of Linking Term Association (LTA), Minimum Weight Association (MWA), and Shared B to C Set Association (SBC), and compare them to Linking Set Association (LSA), concept embeddings vector cosine, Linking Term Count (LTC), and direct co-occurrence vector cosine. Our proposed indirect association measures extend traditional association measures to quantify indirect rather than direct associations while preserving valuable statistical properties.

**Results:**

We perform a comparison between several different hypothesis ranking methods for LBD, and compare them against our proposed indirect association measures. We intrinsically evaluate each method’s performance using its ability to estimate semantic relatedness on standard evaluation datasets. We extrinsically evaluate each method’s ability to rank hypotheses in LBD using a time-slicing dataset based on co-occurrence information, and another time-slicing dataset based on SemRep extracted-relationships. Precision and recall curves are generated by ranking term pairs and applying a threshold at each rank.

**Conclusions:**

Results differ depending on the evaluation methods and datasets, but it is unclear if this is a result of biases in the evaluation datasets or if one method is truly better than another. We conclude that LTC and SBC are the best suited methods for hypothesis ranking in LBD, but there is value in having a variety of methods to choose from.

## Background

### Introduction

Literature Based Discovery (LBD) [1] seeks to find information that is implicit in text, but never explicitly stated. New knowledge can be formed by piecing together fragments of information found across multiple documents. For example, one document may state that “A implies B” and another that “B implies C”; new knowledge is generated by hypothesizing that therefore “A implies C”. In its simplest form, a hypothesis is an assertion that a relationship exists between two terms that never directly co-occur, and the likelihood of that hypothesis being true can be estimated by the strength of their relatedness. In modern LBD systems, hypothesis generation is often more complex and varied than the simple ABC paradigm we used as an example, but there is a critical need for effective hypothesis ranking; both for eliminating uninteresting hypotheses, and for ordering results when displayed to the user. All methods in this paper use term-term pairs to represent and rank hypotheses, and are therefore usable by nearly all LBD systems.

Association measures have been shown to be good indicators of semantic relatedness. They are statistical methods based on two term’s individual and shared co-occurrence frequencies, but they were designed for terms that directly co-occur. In this paper, we present indirect association measures, which incorporate connecting term information to quantify the relatedness between terms that never directly co-occur. Specifically, in this paper we introduce the indirect association measures of:

1. Linking Term Association (LTA), which quantifies association using counts of unique connecting terms
2. Minimum Weight Association (MWA), which quantifies association using co-occurrence counts of A-B-C pathways
3. Shared B to C Set Association (SBC), which quantifies association using the set of shared B terms as a proxy for A

We compare these new methods against Linking Set Association (LSA) [2], concept embeddings cosine [3], linking term count (LTC) [4], and direct co-occurrence vector cosine [5]. We introduce the use of estimating semantic relatedness on standard evaluation datasets (MiniMayoSRS and UMNSRS) as an intrinsic evaluation method for hypothesis ranking in LBD, and we perform extrinsic evaluation using precision and recall (PR) curve analysis [6]. LBD hypothesis ranking methods should perform well for both intrinsic and extrinsic evaluations, and our analysis shows that LTC and SBC are the best performing of the ranking methods evaluated.

This paper begins with a brief overview of related works, which include: ranking methods for LBD, ranking method evaluation, and semantic similarity and relatedness. Next, the methods section begins by describing our implementations of baseline methods, describing traditional association measures, and presenting each indirect association measure in detail. Next, the evaluation methods, datasets, and experimental details are presented Lastly results are shown, and conclusions are made.

### Related work

#### Ranking methods for literature based discovery

The number of hypotheses generated by LBD systems is usually too large to be manually reviewed, so ranking them is critical. One of the first developed, and best performing ranking methods is Linking Term Count (LTC) [4], which counts the number of unique linking (*B*) terms between the start (*A*) and each target (*C*) term. LTC is a purely frequency-based metric, and in an effort to reduce its bias towards frequently occurring terms, several methods that account for both single term occurrence and term-term co-occurrence rates were created. Average Minimum Weight (AMW) [7, 8] calculates the mean of minimum mutual information from *A* to *B* and *B* to *C* for all *A* to *B* to *C* pathways. X to Z support [9] sums the weights of all *ABC* pathways between *A* and *C*, and uses the data-mining metric of support as a weight, but it is noted that other metrics may be used. A more application specific method is Predicate Interdependence [10] which ranks drug-disease pairs based on drug-gene and gene-disease predicate independence versus interdependence in literature. Yetisgen-Yildiz and Pratt [8] perform a comparison between several ranking methods, including LTC and AMW, and find that LTC is best performing hypothesis ranking method evaluated. Due to this performance, we use LTC as a baseline measure for comparison.

Vector-based ranking methods have also been used in LBD. In these cases, a term or concept vector representation is constructed, and a score is generated using cosine distance [11], Euclidean distance [12], or information flow [12] between the *A* and *C* terms. The method in which vectors are created varies by LBD system. Bruza et al. [12] construct vectors in Hyperspace Analogue to Language (HAL) space, Cohen, et. al [13] construct vectors using Predication-Based Semantic Indexing (PSI), and Sybrandt et al. [6] construct vector representations using FastText [14] (a word2vec implementation). We construct word2vec concept embedding vectors and direct co-occurrence vectors, and use cosine distance between the *A* and *C* vectors in our evaluation.

Graph-based ranking methods have also been used. Graph-based methods construct co-occurrence graphs, and rank hypotheses based on the graphs’ characteristics, such as degree centrality [15], or graph proximity metrics such as probability of best path, network reliability, expected reliable distance, or variations of random walks [16]. More recently, Kastrin et al. [17] using the graph proximity metrics of Jaccard’s Coefficient - a ratio of common neighbors to total neighbors, and Adamic/Adar metric - which uses weighted counts of shared neighbors, such that lower connected neighbors receive a higher weight. Sybrandt et al. [6] propose and evaluate several ranking methods for LBD which include concept embeddings, topic network graph based metrics, and a combination of these methods. We compare against their best performing method, PolyMultiple in our extrinsic precision and recall curve evaluation.

#### Ranking method evaluation

The variety of LBD systems [4, 18–25] and the lack of standard evaluation datasets and methodologies have made comparing LBD ranking methods difficult. Evaluation methods have been criticized as too narrow, as is the case with discovery replication [26], too noisy, as is the case with time slicing [8], not quantitative or replicable, as is the case with new discovery proposal [4], or are system specific and do not generalize [23, 27, 28]. Methods that are applicable across systems, quantifiable, and replicable are preferred, and since time slicing and link prediction type evaluation methods look at the presence or absence of links, rather than whether they are in-fact true and novel discoveries they are more easily assessed quantitatively [29].

Time-slicing evaluation was first proposed by Hristovski et al. [20], and later elaborated by Yetisgen-Yildiz and Pratt [8]. It is an evaluation method in which a dataset is divided into pre- and post-cutoff segments, and all post-cutoff co-occurrences or relationships that do not occur in the pre-cutoff dataset are used to estimate future knowledge.

Using co-occurrence information instead of relationship information to estimate future knowledge will capture the greatest number of possible future relationships, but will also capture many false future relationships. This creates a dataset with high recall and low precision [30]. Yetisgen-Yilidiz and Pratt [8] use co-occurrence information to constitute relationships in the pre- and post-cutoff segments, and use precision and recall curves to evaluate several LBD target term ranking measures.

Using relationship information rather than co-occurrence information will capture fewer future relations, but those found will be more accurate, meaning the dataset will have lower recall, but higher precision. Eronen et al. [16] evaluate their system, BIOMINE as a link prediction task. They define link prediction as “the prediction of relationships that are not obvious in the existing data”. Using a biological network of protein interactions and gene-pairs, they select 500 positive (links that are added in a post-cutoff dataset) and 500 negative (links that do not exist in the pre- or post-cutoff datasets) links to generate ROC curves. Sybrandt et al. [6] generalize this idea, and divide a dataset of SemRep predications into pre- and post-cutoff segments, and create three datasets, *highly-cited*, *published*, and *noise*. They view LBD ranking and thresholding as a noise discrimination task, and create *published* versus *noise* and *highly-cited* versus *noise* ROC curves. We use both co-occurrence-based and relationship based time-slicing datasets. We generate a co-occurrence based time-slicing dataset in the same manner as Yetisgen-Yildiz and Pratt [8], and use Sybrandt et al. evaluation dataset to create precision and recall (PR) curves for our LBD extrinsic evaluation.

#### Semantic similarity and relatedness

The likelihood of a hypothesis in LBD being true can be estimated by the strength of the relatedness between the start and target terms. We introduce the use of semantic similarity and relatedness as an intrinsic evaluation method for hypothesis ranking in LBD. Semantic similarity and relatedness measures quantify how similar or related two concepts are. Two terms are related if any relationship exists between them (e.g. aspirin-headache). Semantic similarity is a subset of relatedness, in which the relationship between them is their similarity, typically an isA relationship (e.g. headache-migraine). These measures are critical for many natural language processing applications, such as clustering of biomedical and clinical documents [31], the development of biomedical terminologies and ontololgies [32], and word sense disambiguation [33]. Evaluation of relatedness measures may be performed extrinsically by applying them to a task (e.g. word sense disambiguation) and determining the performance, or intrinsically using several standard evaluation datasets [34, 35]. We use the standard evaluation datasets of UMNSRS [35] and MiniMayoSRS [34] as an intrinsic evaluation method for LBD ranking measures.

## Methods

In this section we describe the LBD hypothesis ranking measures that we evaluate. This includes the baseline measures we use, an introduction to direct association measures, and a detailed explanation of each indirect association measure. These methods rely on co-occurrence data collected from a corpus, and in our implementation, we use the MetaMapped MEDLINE baseline, a corpus of text mapped to United Medical Language System (UMLS) concepts. Each method is, however easily adapted to other data sources by using word or term co-occurrences in place of concept co-occurrences. Similarly, relationship data extracted from a corpus (such as SemRep predications [36]) may be used as a data source by treating the existence of a relationship as a co-occurrence. For this reason, and for clarity, when describing each method, we say “term” to refer to the co-occurrence or relationship between any “concept”, “term”, or “word”.

### Baseline methods

Evaluation between LBD systems and their hypothesis ranking methods is difficult, due to the variety of LBD systems and datasets. In our intrinsic and extrinsic evaluation, only the hypothesis ranking method is evaluated, which means we can evaluate ranking methods regardless of how hypotheses are generated, or from what data-source they are generated from. This allows us to compare different hypothesis ranking methods to our newly proposed indirect association measure ranking methods. These previously proposed methods include linking term count (LTC) [4], direct co-occurrence vector cosine [5], and concept embeddings cosine [3]. For the extrinsic LBD evaluation we compare against Sybrandt et al. [6] PolyMultiple method. Each method is described below.

**Linking Term Count:** We use our own implementation of Linking Term Count (LTC) as a baseline measure in this paper. A linking term is defined as any term for which both *A* and *C* co-occur with, and LTC is defined as the count of linking terms between *A* and *C*. *A* and *C* term pairs with more linking term are ranked higher than those with less in both intrinsic and extrinsic evaluations.

**Cosine Distance:** We use the cosine distance between two different vector representations as baseline measure. These vectors are constructed in a manner similar to Henry et al. [5]. (1) Direct co-occurrence vectors have a dimensionality the size of the vocabulary. Each term in the vocabulary is assigned an index, and each vector contains the co-occurrence count between the term at that index, and the term represented by the vector. The result is a vector containing the counts of all directly co-occurring terms. (2) Concept embeddings are reduced dimensionality distributional context vectors constructed by iterating over a training corpus, and learning concept representations in a neural network based approach. The neural network learns a series of weights (the hidden layer within the neural network) that maximize the probability of a word given the surrounding context. The resulting hidden layer consists of a matrix where each row corresponds to the word embedding for each word in the vocabulary. For both vector representations, we rank terms as the cosine distance between *A* and *C* term vectors.

**PolyMultiple:** In their development of the extracted-relationship based time-slicing dataset, Sybrandt et al. [6] generate Receiver Operating Characteristic (ROC) curves to compare several LBD target term ranking methods. We compare against their best performing metric, PolyMultiple. PolyMultiple is a linear combination of all their evaluated metrics, which include vector similarities, topic model correlations, topic model centroid similarities, and topic model network based methods. Since we use PR curves, rather than ROC curves in our evaluation, we do not show the results, but instead report just the area under the ROC curve (AUROC) to compare against these measures.

### Association measures

**Fig. 1:**
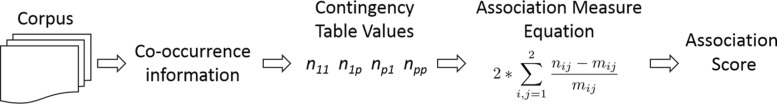
Process overview. An overview of how association measures are calculated. The equation shown is Pearson’s Chi Squared, where *m*_*ij*_ values are: $m_{11} = \frac {n_{1p} * n_{p1}}{n_{pp}}$, $m_{12} = \frac {n_{1p} * n_{p2}}{n_{pp}}$, $m_{21} = \frac {n_{p1} * n_{2p}}{n_{pp}}$, $m_{22} = \frac {n_{p2} * n_{2p}}{n_{pp}}$ and *n*_2*p*_=*n*_*pp*_−*n*_1*p*_, *n*_*p*2_=*n*_*pp*_−*n*_*p*1_

In this section we describe traditional, direct association measures, and introduce our new, indirect association measures. Both direct and indirect association measures are based on co-occurrence statistics in a corpus. They quantify two terms’ expected co-occurrence together by chance versus their observed co-occurrence together in text. Both direct and indirect association measures follow a similar calculation process, which is shown in Fig. 1. In this process, co-occurrence information is collected from a corpus and used to populate a contingency table of values.

Table 1 shows a contingency table for the generic term pair *X* and *Y*, and uses the standard notation, $\overline {X}$ and $\overline {Y}$ to indicate any token except *X* or *Y* respectively. ∗ indicates any single token. The cell *n*_11_ is the joint frequency of the term pair *XY*, the number of times token *X* precedes *Y*. The cell *n*_12_ is the frequency in which *X* occurs in the first position but *Y* does not occur in the second position, and the cell *n*_21_ is the frequency in which *Y* occurs in the second position, but *X* does not occur in the first position. The cell *n*_22_ is the frequency in which neither *X* nor *Y* occur in their respective positions. The cells, *n*_1*p*_, *n*_*p*1_, *n*_2*p*_ and *n*_*p*2_ represent the marginal totals which are the number of times a term does not occur in the first or second position of the term pair. Lastly, the cell *n*_*pp*_ is the total number of term pairs found in the corpus. It is important to note that all contingency table values can be calculated as sums and differences between just four values, *n*_11_, *n*_1*p*_, *n*_*p*1_, and *n*_*pp*_.

**Table 1:** A contingency table showing how the counts, *n*_*xy*_ are calculated for the generic term pair *XY*

	*Y*	$\overline {Y}$	totals
*X*	*n*_11_=*XY*	$n_{12} = X\overline {Y}$	*n*_1*p*_=*X*∗
$\overline {X}$	$n_{21}= \overline {X}Y$	$n_{22} = \overline {X}\overline {Y}$	$n_{2p} = \overline {X}*$
totals	*n*_*p*1_=∗*Y*	$n_{p2} = *\overline {Y}$	*n*_*pp*_=**

$\overline {X}$ and $\overline {Y}$ indicate any token except *X* or *Y* respectively. ∗ indicates any single token

The contingency table values are input into an association measure equation, such as Log Likelihood Ratio [37], Dice Coefficient [38], or Pearson’s Chi-Squared [37] (shown in Fig. 1) to produce a single number that quantifies the association between two terms. To develop indirect association measures, we modify how the contingency table values are calculated prior to input into the association measure equation. The modifications make it possible to quantify the association between indirectly related terms while preserving the beneficial statistical properties encoded in association measure equations. We modify the contingency table values as follows:

1. **Minimum Weight Association (MWA):** modifies *n*_11_ as the average minimum co-occurrence for each A-to-B-to-C pathway
2. **Linking Term Association (LTA):** uses the counts of unique linking terms to populate the contingency table
3. **Shared B to C Set Association (SBC):** uses the co-occurrences between the shared *B* term set and *C* to populate the contingency table
4. **Linking Set Association (LSA):** uses co-occurrences between terms that co-occur with *A* (*B*_*A*_) and terms that co-occur with *C* (*B*_*C*_) to populate the contingency table

MWA and LTA are similar, since rather than using direct *A* to *C* co-occurrences, they combine *A* to *B* and *B* to *C* co-occurrence information. MWA uses *A*−*B* and *B*−*C* co-occurrence counts, while LTA uses the count of unique *B* terms. SBC and LSA are similar, since they are based on set associations. SBC uses the shared *B* terms set as a proxy for *A* when collecting co-occurrence counts, and LSA uses *B*_*A*_ and *B*_*C*_ as proxies for *A* and *C* respectively when collecting co-occurrence counts.

In the next few subsections, we give detailed explanations on how each association measure is calculated. We define the following terminology: *A* is the set of starting term(s); *B*_*A*_ is the set of terms preceded by *A* (*A*’s connecting terms); *C* is the set of target terms; *B*_*C*_ is the set of terms preceding *C* (*C*’s connecting terms); *V* is the set of all terms (the vocabulary); *w*_*ij*_ is the weight of the edge going from node *i* to node *j* in the co-occurrence graph, and is the frequency that term *i* is followed by term *j*.

**Fig. 2:**
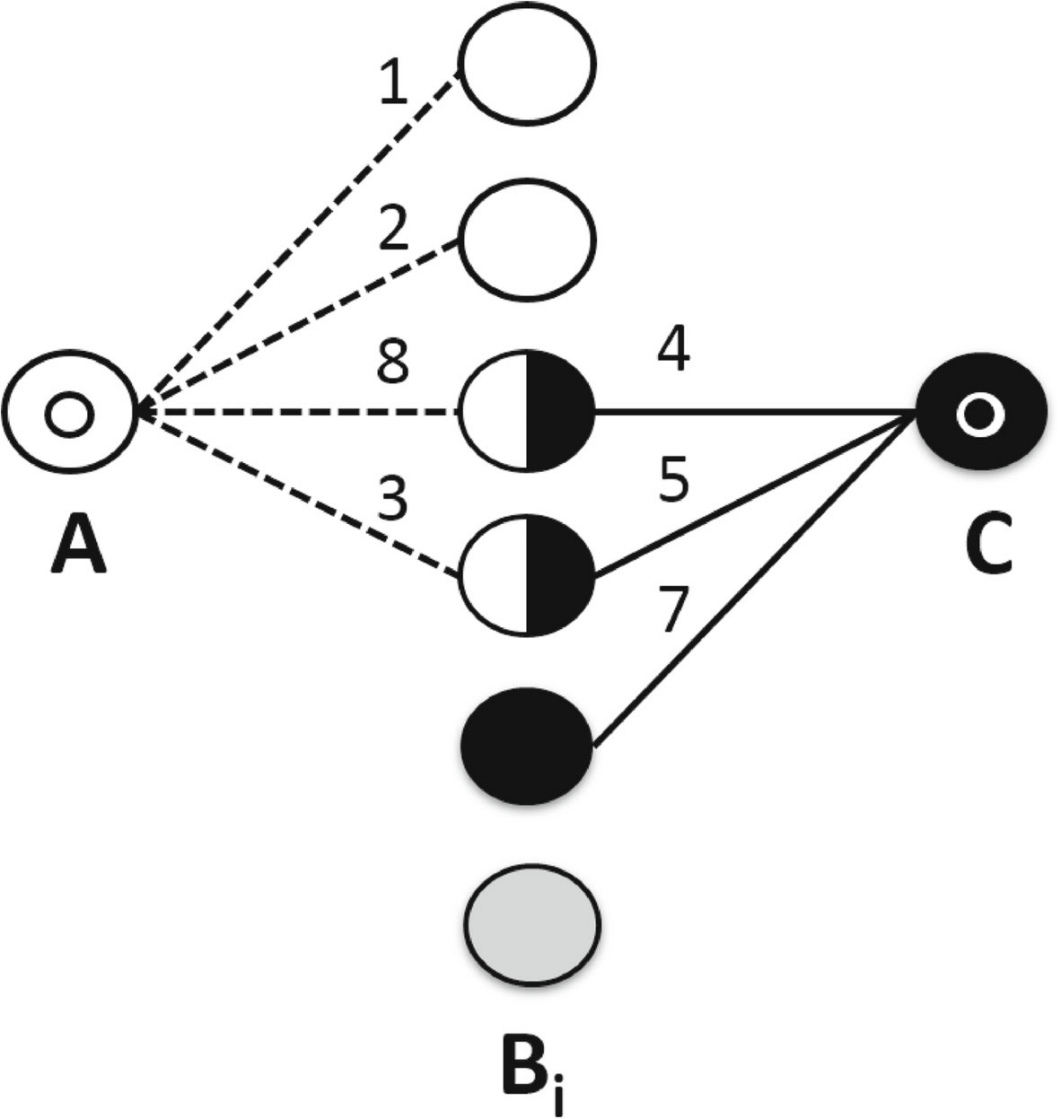
Association scenario. A co-occurrence graph showing *A* and *C* co-occurrences with a set of *B* terms

We also use Fig. 2 as an example. Figure 2 shows a co-occurrence graph between term *A* and term *C*. *A* and *C* directly co-occur with a set of connecting terms, *B*. A subset of which co-occur with both *A* and *C*; an indirect relationship is created through this set of shared *B* terms. Each circle in Fig. 2 indicates a unique term, and each edge indicates a direct co-occurrence. The number above each edge indicates that edge’s co-occurrence frequency. The terms *A* co-occurs with are shown as white and black-and-white circles. The terms *C* co-occurs with are shown as black and black-and-white circles. White *B* terms co-occur with *A* only, black *B* terms co-occur with *C* only, and black-and-white terms are shared linking terms that co-occur with both *A* and *C*. Gray *B* terms exist in the vocabulary, but do not co-occur with *A* or *C*. Figure 2 therefore shows that *A* co-occurs with four different terms, it co-occurs with the top-most *B* term one time, the second top-most *B* term two times, the B term below that eight times, and the term below that three times. *C* co-occurs with three different terms, four times, five times, and seven times respectively, and there is a single *B* term that co-occurs with neither *A* or *C*.

### Direct association measures

Direct association measures have been shown to perform very well at quantifying relatedness [39], but they are unsuitable for LBD hypothesis ranking because they were designed for two terms that directly co-occur. They form the basis for indirect association measures. Using Fig. 2 as an example, we define direct association contingency table values as follows:

*n*_11_ (Eq. 1) is the sum of weights (co-occurrences) between *A* and *C*. In Fig. 2, *n*_11_=0, since A and C never directly co-occur.

1 $$ n_{11} = \sum\limits^{a\in A}{\sum\limits^{c\in C}}{w_{ac}}\\   $$

*n*_1*p*_ (Eq. 2) is the sum of weights between *A* and each *B*_*i*_. In Fig. 2, *n*_1*p*_=1+2+8+3=14.

2 $$ n_{1p} =\sum\limits^{a\in A}{\sum\limits^{j\in V}{w_{aj}}}\\   $$

*n*_*p*1_ (Eq. 3) is the sum of weights between each *B*_*i*_ and *C*. In Fig. 2, *n*_*p*1_=4+5+7=16.

3 $$ n_{p1} = \sum\limits^{j\in V}{\sum\limits^{c\in C}{w_{jc}}}\\   $$

*n*_*pp*_ (Eq. 4) is the sum of all weights in the dataset, which is the total number of co-occurrences between all terms.

4 $$ n_{pp} = \sum\limits^{i\in V}{\sum\limits^{j\in V}{w_{ij}}}   $$

Using these four contingency table values, we can calculate the rest of the values in a contingency table, and calculate an association measure equation, such as Pearson’s Chi Squared (shown in Fig. 1) to test for association between *A* and *C* with a single value. Start-target term pairs in LBD explicitly do not co-occur, meaning *n*_11_=0 for all start-target term pairs. This is shown in Fig. 2, where *A* and *C* never co-occur. Since there is no direct co-occurrence between the terms, we develop indirect association measures which incorporate additional information to quantify the association between two terms which do not directly co-occur, but are instead, indirectly related.

### Minimum weight association

Minimum Weight Association (MWA) calculates association between *A* and *C* based on the information flow between them relative their co-occurrences with all terms in the dataset. It uses co-occurrence counts to populate the contingency table, however we modify the value of *n*_11_ to allow indirect associations to be quantified. We can view each *A* to *B*_*i*_ to *C* link as a weighted path connecting *A* and *C*, and use the co-occurrence information along this path to calculate *n*_11_. The question becomes how to combine the *A*−*B*_*i*_ and *B*_*i*_−*C* weights. One approach may be to sum, average, or take the maximum value of weights along a path, but association measures require that *n*_11_≤*n*_1*p*_≤*n*_*pp*_ and *n*_11_≤*n*_*p*1_≤*n*_*pp*_; sums, averages, or maximums may violate this. Therefore, for MWA we take the minimum value along each *A*−*B*_*i*_−*C* path, and sum over all *A*−*B*_*i*_−*C* pathways. If we imagine the co-occurrence counts along each *A*−*B*_*i*_−*C* pathway as information flowing between *A* and *C*, then summing the co-occurrence counts is analogous to finding the total information flow between *A* and *C*, where each *A*−*B*_*i*_−*C* pathway cannot carry more than its minimum capacity. *n*_1*p*_, *n*_*p*1_, and *n*_*pp*_ remain unchanged from direct association measures. The contingency table values are defined formally as:

*n*_11_, (Eq. 5) is the sum of minimum *A* to *B*_*i*_ and *B*_*i*_ to *C* weights of each shared *B*_*i*_ term. In Fig. 2, *n*_11_=*min*(8,4)+*min*(3,5)=7.

5 $$ n_{11} = \sum\limits^{b\in B_{A} \cap B_{C}}{min\left(\sum\limits^{a\in A}{w_{ab}}, \sum\limits^{c\in C}{w_{bc}}\right)}   $$

*n*_1*p*_, (Eq. 2) remains unchanged from direct association measures. It is the sum of *A* to *B*_*i*_ weights. In Fig. 2, *n*_1*p*_=1+2+8+3=14.

*n*_*p*1_, (Eq. 3) remains unchanged from direct association measures. It is the sum of *B* to *C* weights. In Fig. 2, *n*_*p*1_=4+5+7=16.

*n*_*pp*_, (Eq. 4) remains unchanged from direct association measures. It is the sum of all possible weights (total co-occurrence count) of the whole dataset.

### Linking term association

Linking term association (LTA) quantifies the association between *A* and *C* based on the count of shared linking terms. It combines the empirically proven performance of Linking Term Count (LTC) with the statistical properties of association measures. Rather than using co-occurrence counts for contingency table values, LTA uses counts of unique co-occurring terms. If we view the co-occurrence graph in Fig. 2 as an unweighted graph, the contingency table value equations for LTA are identical to MWA, but it is perhaps more intuitive to define these values in terms of set theory.

*n*_11_, (Eq. 6) is the count of unique shared linking terms. In Fig. 2, *n*_11_=2.

6 $$ n_{11} = |B_{A} \cap B_{C}|   $$

*n*_1*p*_, (Eq. 7) is the count of unique terms *A* co-occurs with. In Fig. 2, *n*_1*p*_=4.

7 $$ n_{1p} = |B_{A}|   $$

*n*_*p*1_, (Eq. 8) is the count of unique terms *C* co-occurs with. In Fig. 2, *n*_*p*1_=3.

8 $$ n_{p1} = |B_{C}|   $$

*n*_*pp*_, (Eq. 9) is the count of all possible unique terms (vocabulary size).

9 $$ n_{pp} = |V|   $$

In this formulation, the value of *n*_11_ is equivalent to the LTC between *A* and *C*, but we weight the LTC by the number of terms *A* and *C* independently co-occur with in the association measure equation. This makes the associations between terms that independently co-occur with many terms lower than those that independently co-occur with a just few terms.

### Shared B to C set association

Shared B to C association (SBC) quantifies the association between *A* and *C* as the set association between their shared *B* terms and *C* itself. It builds upon the idea of set associations [2], which quantify the association between sets of terms rather than individual term-term pairs. SBC uses the shared *B* terms between *A* and *C* as a proxy for the *A* term, then calculates the direct set association between that proxy and *C* itself. That is, we populate the contingency table using the co-occurrences between the shared *B* term set and *C*, rather than between *A* and *C*. Equation 10 formally defines SBC in terms of set theory, where *assoc*() is an association measure equation such as Pearson’s Chi Squared (shown in Fig. 1).

10 $$ assoc(B_{A} \cap B_{C}, C)   $$

### Linking set association

Linking Set Association (LSA) quantifies association between *A* and *C* using the set association between *B*_*A*_ and *B*_*C*_. Like SBC, LSA is based on direct associations between sets of terms, but for LSA, we replace both *A* and *C*. *A* is replaced with the set of all terms that it co-occurs with (*B*_*A*_), and *C* is replaced with the set of all terms that it co-occurs with (*B*_*C*_). The association between these two sets is then calculated.

11 $$ assoc(B_{A}, B_{C})   $$

Intuitively, both SBC and LSA are estimating *A* and *C* with a set of terms. LSA estimates *A* and *C* with their co-occurrences, which is indicative of their context, and therefore their meaning. SBC estimates *A* with respect to its shared co-occurrences with *C*, and uses *C* directly. In other words, the association between how *A*’s meaning overlaps with *C*’s meaning, and *C* itself. LSA defines its proxies in terms of their own independent contexts, where as SBC defines the proxies in terms of the more constrained, shared context.

### Evaluation

We evaluate the proposed indirect association measures both intrinsically on the task of estimating semantic similarity and relatedness, and extrinsically on the task of ranking target terms for LBD. Each evaluation technique measures a different aspect of performance:

1. Estimating similarity and relatedness - uses human-generated gold standard datasets of term pairs that directly co-occur to evaluate the ability of a measure to estimate how similar or related two terms are.
2. Ranking target terms for LBD - uses automatically-generated silver standard datasets of term pairs that do not co-occur to evaluate the ability of a measure to rank target terms in LBD.

Since it is our hypothesis that ranking in LBD should be based on estimating the strength of a relationship between two unrelated terms, ranking methods should perform well at estimating semantic relatedness for two terms regardless of if they co-occur together or not. Estimating semantic relatedness is a well established field with standard, human-generated gold standard evaluation datasets. We use these datasets in our evaluation, but they contain terms that directly co-occur, which does not evaluate the ability of a measure to estimate relatedness between terms that never co-occur, as is the case with start-target term pairs in LBD.

We therefore also evaluate on the extrinsic task of ranking target terms for LBD, for which evaluation methods and datasets are less standardized. We use time-slicing based techniques to automatically create silver standard datasets. The silver standard datasets consist of start-target term pairs representing both true and false future discoveries. We estimate this using time-slicing, in which a dataset is divided into pre- and post-cutoff segments. The post-cutoff segment estimates future knowledge, and use the pre-cutoff segment estimates known knowledge. Term pairs that occur in the post-cutoff segment, and not in the pre-cutoff segment represent new discoveries, and form the true samples of the silver standard dataset. Their is no best method to generate a time-sliced dataset, so we evaluate using two silver standard datasets. One based on term co-occurrences, and another based on extracted relationships. We describe the intrinsic evaluation method and datasets, and both extrinsic evaluation method and datasets in the next subsections.

#### Semantic similarity and relatedness intrinsic evaluation

Intrinsic evaluation is performed by estimating semantic similarity and relatedness. It is our hypothesis that ranking in LBD should be based on estimating the strength of a relationship between two terms, and ranking methods should therefore, perform well at estimating semantic relatedness. We evaluate using the reference standards of: the UMNSRS [35] tagged for similarity (UMNSRS Sim), the UMNSRS tagged for relatedness (UMNSRS Rel), the MiniMayoSRS dataset [34] rated by medical coders (MiniMayo Cod) and the MiniMayoSRS rated by physicians (MiniMayo Phys). We report Spearman’s Rank Correlation Coefficient (*ρ*) between the scores generated for each term pair and these gold standards scores.

MiniMayoSRS consists of 30 term pairs whose relatedness was determined by nine medical coders and three physicians from the Mayo Clinic. The relatedness of each term pair was assessed based on a four point scale: (4.0) practically synonymous, (3.0) related, (2.0) marginally related and (1.0) unrelated. MiniMayoSRS is a subset of the MayoSRS [34], for which a high inter-annotator agreement was achieved. The average correlation between physicians is 0.68. The average correlation between medical coders is 0.78. We evaluate our method on the mean of the physician scores, and the mean of the coders scores on the 29 term pairs found within the Systematized Nomenclature of Medicine - Clinical Terms (SNOMED CT) terminology [40].

UMNSRS, developed by Pakhomov et al. [35], consists of 725 clinical term pairs whose semantic similarity and relatedness was determined independently by four medical residents from the University of Minnesota Medical School. The similarity and relatedness of each term pair was annotated based on a continuous scale by having the resident touch a bar on a touch sensitive computer screen to indicate the degree of similarity or relatedness. As suggested by Pakhomov and colleagues, we use a subset of ratings with higher Intraclass Correlation Coefficients (ICCs). This subset has an ICC of 0.73, and consists of 401 pairs for the similarity set, and 430 pairs for the relatedness set.

Some examples of concept pairs in these evaluation datasets are: difficulty walking, antalgic gait (C0311394, C0231685); rheumatoid nodule, lung nodule (C0035450, C0034079); hand splint, splinter hemorrhage (C0409162, C0333286); diabetes, polyp (C0011849, C0032584); and portal hypertension, nevus (C0020541, C0027962).

The MiniMayoSRS and UMNSRS datasets contain term pairs of different UMLS semantic groups, concept pairs of the same semantic group, and synonymous term pairs. We can further analyze the performance by selecting concept pairs most relevant to LBD. The UMNSRS dataset contains concepts from primarily from the semantic groups of *Disorders* and *Chemicals and Drugs*. There are 113 and 126 *Disorders*-*Chemicals and Drugs* or *Chemicals and Drugs*-*Disorders* concept pairs in the UMNSRS Sim and Rel datasets respectively [41]. Since LBD is often applied to finding new treatments (chemicals and drugs) for diseases and disorders, these concept pairs are particularly relevant for LBD and can be used to better evaluate the target term ranking algorithms’ performance. We report results using both the full datasets for comparison between other papers, and also on this subset, which we recommend using alone for use in future LBD target term ranking evaluation.

#### Target term ranking for LBD extrinsic evaluation

Extrinsic evaluation for ranking terms in LBD is performed by using time-slicing techniques in a manner similar to that outlined by Yetisgen-Yildiz and Pratt [8]. Both ROC curves [6, 16, 17] and PR curves [8] have been used as a time-slicing evaluation methods. PR curves and ROC curves show similar information. An ROC curve shows the true and false positive fractions on each axis, where as a PR curve shows the precision and recall on each axis. PR curves have been shown to be more informative for tasks with a severe class imbalance [42], such as LBD, so we use PR curves in our evaluation.

To generate a PR curve, we use time-slicing evaluation in which a silver standard evaluation dataset is created by dividing a dataset into pre- and post-cutoff segments. The silver standard contains true and false term pairs. The true term pairs are created by finding term pairs that occur in the post cutoff segment and do not occur in the pre-cutoff segment. False term-term pairs are created as pairs that occur in neither the pre- or post-cutoff segments. Data from the pre-cutoff segment is used to calculate scores for each silver standard term pair, and the pairs are ranked in descending order, meaning pairs with high scores have a high estimated semantic relatedness, and pairs with low scores have a low estimated semantic relatedness. A PR curve is generated by applying a threshold at each rank. Pairs ranked below the threshold are considered false, and pairs above are considered true. The precision and recall at each rank is calculated and plotted to create a PR curve, and the area under the curve (AUC) may be calculated to quantify performance with a single number. To penalize methods that produce term pairs with tied rankings, we penalize tied pairs by always ranking false pairs higher than true pairs in the event of a tie.

An ideal silver standard would contain all possible future discoveries, and no currently known discoveries. This is an impossibility, and there is no widely accepted silver standard dataset, and no consensus on the best way to generate it. To address this in our extrinsic evaluation, we use two silver standard datasets, each with different strengths and weaknesses. One is based on co-occurrence information, and the other is based on extracted-relationship information.

### Time-slicing datasets

We use two silver standard datasets in our extrinsic evaluation. These datasets both simplify future discoveries as future relationships, and a future relationship is simplified as a term pair. Term pairs that occur in the post-cutoff dataset and not in the pre-cutoff dataset are considered new discoveries. The datasets differ in how the pre- and post-cutoff pairs are generated. One uses co-occurrence information, and the other uses extracted relationships. Each method has advantages and disadvantages, which we contrast using the following characteristic desirable in a time-slicing dataset:

1. Representative - have statistical properties similar to real-world LBD data
2. High Precision - contain minimal false discoveries
3. High Recall - contain maximum true discoveries

For our evaluation, we create a co-occurrence based dataset as outlined by Yetisgen-Yildiz and Pratt [8], and use an extracted-relationship based dataset developed by Sybrandt et al. [6]. Both datasets use Unified Medical Language System (UMLS) concept pairs instead of term pairs to represent relationships, but a mapping between terms and concepts exists (and can be found using a tool such as the UMLS-Interface [43]), so these datasets can be used regardless of whether a system uses concepts or terms. Both datasets are imperfect, but we relax the constraint that the silver standard datasets must contain all possible future discoveries, and instead evaluate based solely on the presence or absence of samples in each dataset, making them more easily assessed [6, 16, 17, 29].

#### Our co-occurrence based dataset:

We create a co-occurrence based time slicing dataset using the procedure outlined by Yetisgen-Yildiz and Pratt [8]. In this dataset, we use co-occurrence information to constitute a relationship. We collect co-occurrence information using UMLS::Association version 1.3’s CUI Collector tool^1^ run over titles and abstracts of the 2015 MetaMapped MEDLINE Baseline, with sentence boundaries ignored. We used a window size of 8 (meaning 8 concepts after a concept are counted) and default values for all other parameters. As with Yetisgen-Yildiz and Pratt [8], we use a cutoff date of January 1, 2000.

The silver standard dataset is constructed using start-target term pairs. Two hundred start terms are selected by randomly choosing 50 terms from each of the semantic types of: Clinical Drug (T200, clnd), Pharmacologic Substance (T121, phsu), Disease or Syndrome (T047, dsyn), Sign or Symptom (T184, sosy). The set of target terms is defined as all terms in the vocabulary, and start-target term pairs are generated for all possible start-target term pairs. Term pairs that exist in the pre-cutoff segment are removed, which results in a labeled silver standard dataset of all possible start-target terms pairs for each of the 200 start terms. Those that occur in the post-cutoff dataset are labeled as true, and those that do not are labeled as false.

This dataset is *somewhat representative* of LBD data, since using all terms in the vocabulary mimics how LBD is performed, the distribution of true and false samples should be representative of real LBD data. It, however, relies of randomly selecting 200 start terms, and there is no guarantee these terms are representative samples. The dataset has *low precision* since using co-occurrence information over-generates relationships [30]. This dataset has *high recall*, since using co-occurrences will capture nearly all true relationships in the data.

Some examples of false concept pairs in this dataset include: sulfadiazine 500mg, sh869 (a derivative of dipyridamole) (C0990411, C0074443); premenstrual symptom, infection caused by leishmania tropica minor(C0232959, C0086541); and recurrent low back pain, asarumin B (C0751648, C0646400). Some examples of true concept pairs in this dataset include: cicatrix of tonsil, age differences (C0272389, C0699810); eruption of skin, melanoma antigen recognized by t cells(C0015230, C1334510); and lower extremity weakness, glucosamine (udp-n-acetyl)-2-epimerase/nacetylmannosamine kinase (C1836296, C1428183).

#### Sybrandt’s extracted-relationship based dataset:

Sybrandt et al. [6] create an extracted-relationship based time-slicing dataset. Their dataset uses extracted relationships to constitute a relationship. They use SemMedDB [44], a database of semantic predications extracted from MEDLINE by SemRep [36]. SemRep [36] extracts relationships from biomedical text as semantic predications in the form of subject-predicate-object triples. For example, *aspirin ASSOCIATED_WITH headache*, where *aspirin* and *headache* are concepts, and ASSOCIATED_WITH is a SemRep relation type. The concept pairs of these predications are used to represent relationships, and they divide SemMedDB into pre- and post-cutoff segments using a cutoff date of January 1, 2010.

They construct two silver standard datasets from three sets of pairs. (1) *Published* pairs, which consists of 4319 concept pairs which occur only in the post-cutoff segment; (2)*Highly cited* pairs, which consists of 1448 concept pairs selected from the *published* dataset which occur in papers that are cited at least 100 times, and (3) *Noise* pairs which do not occur in the pre- or post-cutoff segments. They create a *published versus noise* silver standard dataset by randomly combining all 4319 *published* pairs with 4319 randomly selected *noise* pairs, and a *highly-cited versus noise* silver standard dataset by randomly combining all 1448 *highly-cited pairs* pairs with 1448 randomly selected *noise* pairs. *Noise* pairs are treated as false, and *published* and *highly-cited* pairs are treated as true.

This dataset is *not very representative* of LBD data. There are two reasons for this: (1) Sybrandt et al. artificially produce a balanced dataset, but since most term pairs are not future discoveries, LBD evaluation datasets should have a high class imbalance; (2) the terms used in their true and false pairs have distinct differences in occurrence rates.

We found this difference in occurrence rates using co-occurrence counts collected from the pre-cutoff (January 1, 2010) segment of MEDLINE. For each term in each term pair, we calculated the number of unique terms it co-occurs with, and its total number of occurrences in the pre-cutoff segment. Table 2 summarizes our findings. It shows the average number of co-occurring terms, and average number of occurrences for the start (*A*) and target (*C*) terms in the *highly-cited*, *published*, and *noise* datasets.

**Table 2:** ROC dataset co-occurrence means

Difference in co-occurrences rates between terms in each dataset		
Term set	Mean co-occurring terms	Mean occurrences
Highly-cited *A*	13,587	987,086
Highly-cited *C*	9065	607,984
Published *A*	10,312	627,894
Published *C*	7109	398,202
Noise *A*	2152	82,555
Noise *C*	1770	76,213

Average number of co-occurring terms and average occurrence count for each *A* and *C* term in the highly-cited, published, and noise datasets

Table 2 shows that on average, both the start (*A*) and target (*C*) terms in the true pair sets (*highly-cited* and *published*) occur much more frequently and co-occur with many more terms than the terms in the false (*noise*) dataset. This difference in occurrence rates is understandable, since highly cited term pairs may come from more popular research areas than just any published term pair, and noise term pairs that never co-occur together likely consist of rarely used terms. This difference in occurrence rates between true and false terms creates a bias in the dataset.

Sybrandt et al. dataset is, however *fairly precise*, since using relationships rather than co-occurrences greatly increases the precision of the extracted relationships. SemRep has precision rates between 73% and 96% [44] depending on the relationship type, and the accuracy of the extracted relationships was found to be 84% [45]. This increase in precision from using SemMeDB also means a *decreased recall*; SemRep recall rates were found to be between 55-70% depending on the relationship type.

Lastly, Sybrandt et al. dataset relies solely on SemMedDB to create the pre- and post cutoff segments, and although the true concept pairs (*highly-cited* and *published* sets) may be absent from the pre-cutoff segment of SemMedDB, they may co-occur together in a pre-cutoff version of MEDLINE. We found that over half of these true pairs directly co-occur in the pre-cutoff portion of MEDLINE. Although this is not ideal, it is acceptable, as long as only SemMedDB data is used to calculate scores in ranking.

Some examples of *highly-cited* concept pairs in this dataset include: mitogen, rett syndrome (C0018284, C0035372); cerebral vascular disorder, grains (C0007820, C0086369); and psoriasis, rituximab antibody (C0033860, C0393022). Some Examples of *published* concept pairs include: carbonyl cyanide chlorophenyl hydrazone, barasthesia (C0007043, C0234222); natural regeneration, lozartan (C0034963, C0126174); and exocytosis, lumen formation in an anatomical structure (C0015283, C1523599); and some examples of *noise* pairs include: filamin binding lim protein, ferm domain-containing protein (C1825283, C1825283); lipanor, epiphysis of tibia (C0591814, C1282300); and montanoas, pediatric pain assessment (C1135607, C1827921).

#### Summary

To summarize, there is no agreed upon best method to create time-slicing datasets, so we use two methods, each with strengths and weaknesses. Our co-occurrence based dataset uses co-occurrence data to constitute relationships. Using co-occurrences means that the silver standard will have a higher recall, but much lower precision. We use all possible start-target term pairs as a silver standard so the class distribution is likely representative of LBD data, but using only 200 randomly could introduce a bias. Sybrandt et al. extracted-relationship based dataset used SemMedDB predications to constitute relationships. Using extracted-relationships means that the silver standard will have lower recall, but much higher precision. They artificially create a balanced class distribution, so the data may not be representative of class distributions in LBD.

### Experimental details

In this section, we describe the specifics of how results were generated. Code and data, including co-occurrence matrices and concept embeddings are available online^2^.

#### Corpus

Each ranking method relies on co-occurrences collected from a corpus. We use the 2015 *MetaMapped MEDLINE baseline*^3^. The MetaMapped MEDLINE Baseline is a database of biomedical and life science journals mapped to United Medical Language System (UMLS) concepts by using the MetaMap tool [46]. Using MetaMapped text has the effect of performing stop word removal and text normalization. For our intrinsic evaluation of estimating semantic relatedness, no time slicing is required, and we use data from January 1, 1975 to December 31, 2015 to construct a co-occurrence matrix. For our co-occurrence based time-slicing dataset, we use a time-sliced version of the 2015 *MetaMapped MEDLINE baseline* for which all data from January 1, 1975 to December 31, 1999 is used to construct a co-occurrence matrix. For Sybrandt et al. [6] extracted-relationship based time-slicing dataset, we use a time-sliced version of SemMedDB version 31_R processed up to June 30, 2018. We use a cutoff date of January 1, 2010. Predications are treated as co-occurrences, and term pairs in predications extracted from publications prior to the cutoff date are used to create a co-occurrence matrix.

#### Co-occurrence matrix

For our intrinsic evaluation of estimating semantic relatedness, and our extrinsic co-occurrence based time-slicing evaluation, we create a co-occurrence matrix in the same manner as Henry et al. [39], who perform a study to optimize several parameters of direct association measures. We use UMLS::Association version 1.3’s CUI Collector tool^4^ run over titles and abstracts of the 2015 MetaMapped MEDLINE Baseline, with sentence boundaries ignored. This tool takes MetaMapped text as input, and treats it as a sequence of UMLS concepts. We used a window size of 8 (meaning 8 concepts after a concept are counted) and default values for all other parameters. The result is a co-occurrence matrix for which each row corresponds to the co-occurrences of a single UMLS concept to every other UMLS concept (indicated by the column). We apply a minimum co-occurrence threshold of 1 to this matrix, meaning all matrix values less than or equal to 1 are set to 0. This removes noise and greatly increases the sparsity of the matrix, reducing computation time with little effect on performance [39]. For Sybrandt et al. extracted-relationship based time-slicing evaluation, MEDLINE co-occurrences are not used, and instead only term pairs in SemMedDB predications are used. The co-occurrence matrices are used to compute all of the indirect association measures (LTA, MWA, SBC, LSA), and LTC. The rows of the matrices are used as vectors for the direct co-occurrence cosine method.

#### Indirect association measures

Indirect association measures and LTC are implemented in the UMLS::Association v1.7 package^5^, a Perl implementation of association measures. LTC is calculated using the ‘- -lta’ option with ‘measure=freq’. The Pearson’s Chi Squared association measure (‘measure =x2’) was selected for the association equation for all indirect association measures, because it has been shown to perform well for semantic similarity and relatedness with direct associations [39].

#### Concept embeddings

Concept embeddings rely on co-occurrence information, but not a co-occurrence matrix. Vector representations are created as the training algorithm iterates over a corpus. For creating concept embeddings, we use abstracts from the 2015 MetaMapped MEDLINE baseline as input into the word2vec-interface package version 0.03^6^ with the Continuous bag of words (CBOW) embedding model, a window size of 8, a frequency cutoff of 0, and default settings for all other hyper-parameters. These hyper-parameters have been shown to perform well when using concept embeddings for semantic similarity and relatedness [5]. The full MEDLINE dataset was used for intrinsic evaluation of estimating semantic similarity and relatedness, and the pre-cutoff MEDLINE segment was used for our co-occurrence based time-slicing evaluation. Concept embeddings are not constructed for evaluation with the Sybrandt et al. dataset, since it is unclear how to best generate them from predication information.

## Results

In this section, we evaluate our indirect association measures (LTA, MWA, SBC, and LSA) against the baselines of concept embedding cosine distance (Emb Cos), direct co-occurrence vector cosine distance (Dir Cos), linking term count (LTC), and randomly assigned scores.

### Semantic similarity and relatedness results

Table 3 shows results for each method on each dataset on the task of estimating semantic similarity and relatedness. Each row shows the results for a single ranking method, and each column for a single dataset. The Spearman’s Rank Correlation coefficient (*ρ*) with the number of terms (n) compared in parentheses are shown for each method on each dataset. The top two rows show the performance of baseline methods of randomly assigning scores and LTC. The middle four rows show results for indirect association scores (LTA, MWA, SBC, LSA), and the bottom two rows show results for vector-based methods (direct cosine (Dir Cos) and embeddings cosine (Emb Cos)). Higher values of *ρ* are better, and indicate higher rank correlation to the gold standard. Higher values of n indicate more terms are able to be compared. The best performing method for each dataset is shown in bold.

**Table 3:** Semantic relatedness results

Correlation Coefficients (*ρ*) and number of samples (n)				
Measure	MiniMayo Cod	MiniMayo Phys	UMNSRS Sim	UMNSRS Rel
Random	-0.0300 (29)	-0.1279 (29)	-0.0185 (401)	-0.0113 (430)
LTC	0.5132 (29)	0.5063 (29)	0.2195 (390)	0.2386 (415)
LTA	0.4930 (29)	0.5403 (29)	0.4772 (390)	0.3526 (415)
MWA	0.2902 (29)	0.3231 (29)	0.3617 (390)	0.2606 (415)
SBC	0.6351 (29)	0.5978 (29)	0.5163 (389)	0.5112 (414)
LSA	0.3881 (29)	0.4027 (29)	0.3366 (390)	0.3080 (415)
Dir Cos	0.5946 (29)	0.5165 (29)	0.5315 (390)	0.4015 (415)
Emb Cos	**0.7762** (29)	**0.6942** (29)	**0.7038** (392)	**0.5537** (418)

Spearman’s Rank Correlation Coefficient and the number of terms compared for each method on the task of semantic similarity and relatedness. Each row shows the results for a single method, and each column for a different dataset. Bold terms indicate the best performance

Emb Cos performs the best for each dataset (MiniMayo Cod., MinMayo Phys., UMNSRS Sim, and UMNSRS Rel), and SBC performs the second best for each dataset except UMNSRS Sim, for which Dir Cos performs better. We calculate *p*-values using Fisher’s R-to-Z transformation [47] to determine statistical significance between these results, and use *p*≤0.05 to indicate statistical significance. Emb Cos performs statistically significantly better than Dir Cos on the UMNSRS Sim and UMNSRS Rel datasets, and statistically significantly better than SBC on only the UMNSRS Sim dataset. SBC performs statistically significantly better than direct cosine on only the UMNSRS Rel dataset. The results of other indirect association measures and LTC are mixed. MWA performs worse than LTA and LSA for all datasets, and worse than LTC for both MiniMayo datasets; it is the worst performing indirect association measure. LTC performs well for the MiniMayo datasets, and poorly for the UMNSRS datasets, indicating that since it is a simplistic method, it may not be able to effectively quantify indirect association for all concepts, and that the concepts in the MiniMayo dataset may be “easy” examples. LTA performs better than LSA for each dataset.

All methods are able to quantify most concepts in all datasets (indicated by n), but only 390 of the 401 UMNSRS Sim concepts and 415 of the 430 UMNSRS Rel concepts. Notably, SBC cannot calculate the association for one less concept than other indirect association measures for the UMNSRS Sim and UMNSRS Rel datasets. When concepts share no linking terms, the shared B to C set is undefined, and association cannot be quantified.

Table 4 shows results for each method on estimating semantic similarity and relatedness for *Disorders* and *Chemicals and Drugs* concept pairs. Each row shows the results for a single ranking method, and each column for a single dataset. The Spearman’s Rank Correlation coefficient (*ρ*) with the number of terms (n) compared in parentheses are shown for each method on each dataset. The best performing method for each dataset is shown in bold.

**Table 4:** Semantic relatedness results for *Disorders* and *Chemicals and Drugs* semantic group pairs

Correlation Coefficients (*ρ*) and number of samples (n)		
Method	UMNSRS Sim	UMNSRS Rel
Random	0.0460 (109)	0.0433 (122)
LTC	0.2480 (109)	0.2190 (121)
LTA	0.1622 (109)	0.3191 (121)
MWA	0.0412 (109)	0.2435 (121)
SBC	0.3639 (109)	0.4146 (121)
LSA	0.1982 (109)	0.2663 (121)
Dir Cos	0.2519 (109)	0.2878 (121)
Emb Cos	**0.5690** (109)	**0.5730** (122)

Spearman’s Rank Correlation Coefficient and the number of terms compared for each method on the task of semantic similarity and relatedness. Each row shows the results for a single method, and each column for a different dataset. Bold terms indicate the best performance

Results for *Disorders* and *Chemicals and Drugs* semantic group pairs are lower than results using the full datasets. This indicates that this is a harder problem. The order of performance of methods is similar to the full dataset. Emb Cos performs the best, and SBC performs the second best for both UMNSRS Sim and Rel subsets. Dir Cos performs the third best, and fourth best for the UMNSRS Sim and Rel datasets respectively. Results are mixed for the other methods, but interestingly, LTC performs third best for the UMNSRS Sim subset, and the worst on the UMNSRS Rel subset. LTA performs third best on the UMNSRS Rel subset, and second worst on the UMNSRS Sim subset. MWA performs very poorly on the UMNSRS Sim subset, but OK on the UMNSRS Rel subset. This may indicate that it is better at estimating relatedness than similarity. Emb Cos performs statistically significantly better than SBC on neither dataset, but statistically significantly better than the third best performing measures (Dir Cos and LSA) on both datasets. Only 109/113, and 122/126 concept pairs for the UMNSRS Sim and UMNSRS Rel subsets occur in our corpus. Only 121/122 concept pairs can be computed using the metrics based on a co-occurrence matrix (LTC, LTA, MWA, SBC, LSA, and Dir Cos), because the concept for prostatorrhea (C0392071) is removed from the co-occurrence matrix when the threshold of 1 is applied.

### Co-occurrence based time-slicing results

**Fig. 3:**
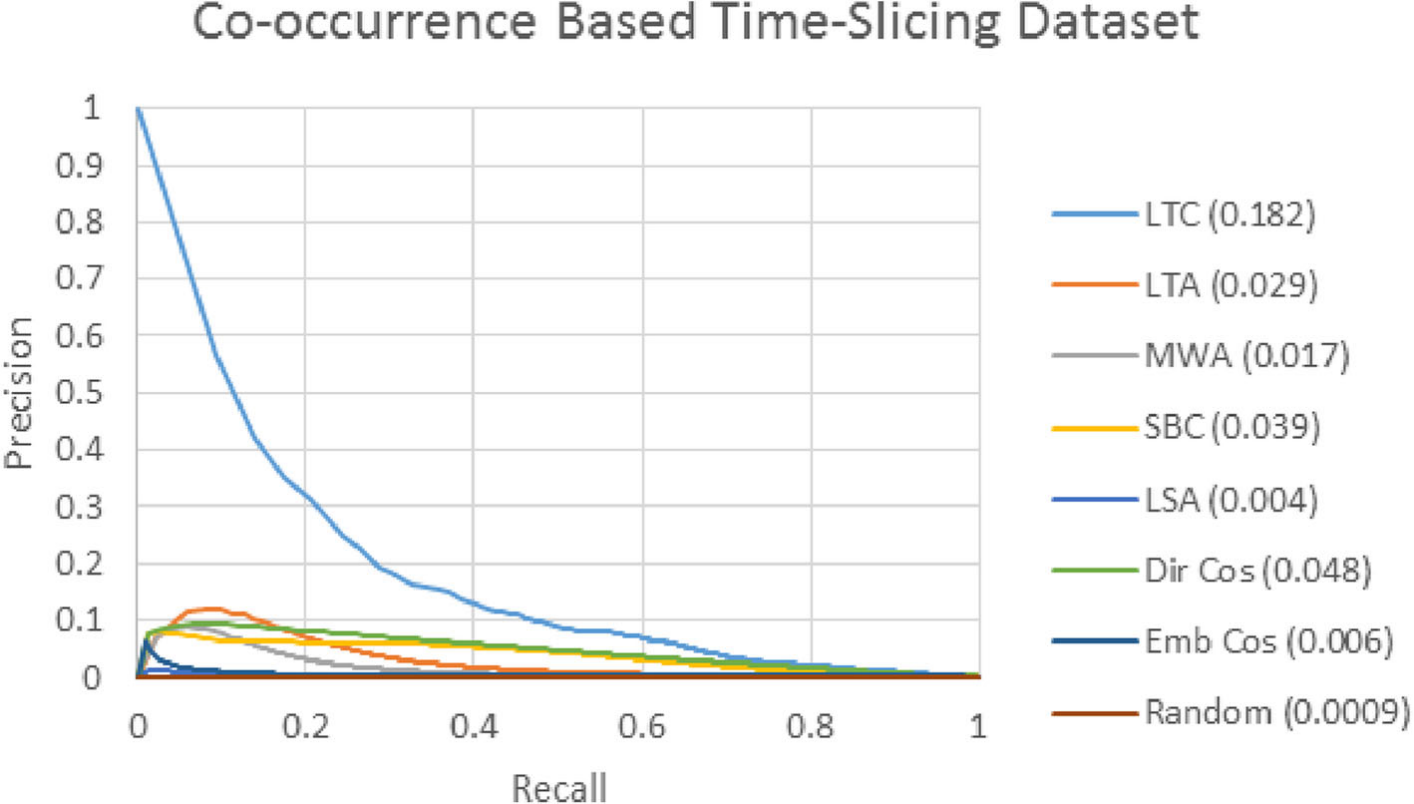
Co-occurrence Dataset PR Curve This ROC curve shows the ability of each ranking method to distinguish between term pairs that newly co-occur in the post-cutoff dataset and those that co-occur in neither the pre- or post cut-off datasets

In this section, we present the results using the co-occurrence based time slicing dataset we created based on the procedure outlined by Yetisgen-Yildiz and Pratt [8]. Figure 3 shows the PR curve. Each colored line corresponds to a different target term ranking method. Figure legends show the area under the curve (AUC) in parentheses.

In PR curve analysis, an ideal classifier would produce a curve that goes straight up the y-axis at a value of 0.0 and straight across the x-axis at a value of 1.0, and produce an AUC of 1.0. A random classifier would produce a line straight across at a value of the true/false class ratio, which in this case is 0.00089. Lines closer to this perfect scenario with higher AUCs are better, and lines closer to this random scenario with lower AUCs are worse. We cast target term ranking as a classification problem by scoring and ranking each concept-concept pair and applying a threshold at each rank. False concept-concept pairs above the threshold are false positives, and the true concept-concept pairs above the threshold are true positives.

**Fig. 4:**
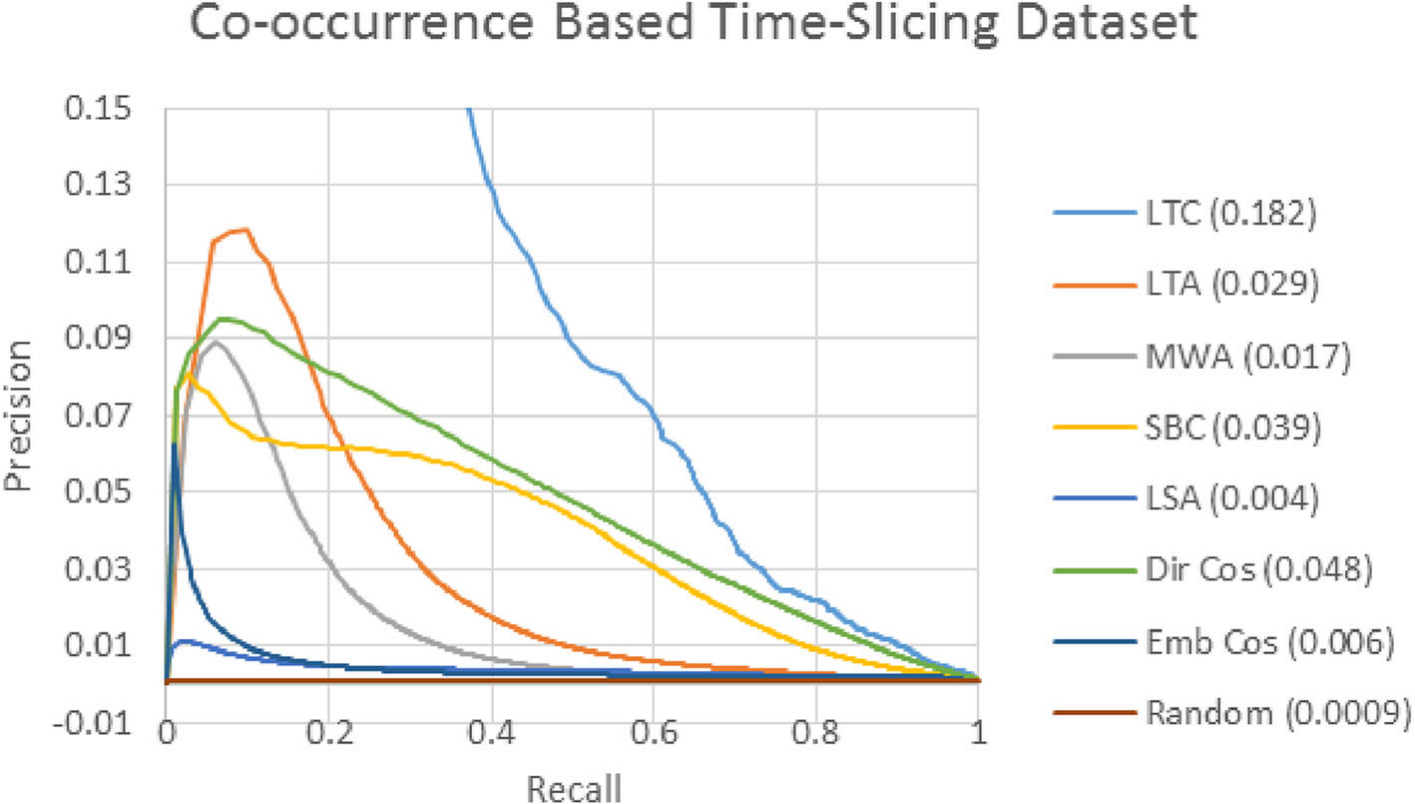
Co-occurrence Dataset PR Curve (zoomed in) This ROC curve shows the ability of each ranking method to distinguish between term pairs that newly co-occur in the post-cutoff dataset and those that co-occur in neither the pre- or post cut-off datasets.. To better distinguish between methods other than LTC, the maximum value on the Y-axis has been changed

LTC is the best performing metric, and is the only one to achieve high precision rates at low levels of recall. This good performance of LTC confirms the findings of Yetisgen-Yildiz and Pratt [8], who also found that LTC performs the best for their co-occurrence based evaluation dataset. Since LTC performs much better than the other methods, it is difficult to distinguish the curves of the other measures from one another, so in Fig. 4, we reduce the maximum value of the Y-axis to better distinguish the other methods from one another.

Figure 4 shows that LTC performs better than all other measures at all levels of recall. Dir Cos performs the second best overall, and performs better than the other methods at most levels of recall, except for LTA which performs better at low levels of recall. LTA and MWA perform better than SBC for low levels of recall, but SBC performs better for higher recall levels. For LBD, where the truth values of the highest ranked terms is important, LTA and MWA may be preferred over SBC on this dataset, even though SBC has a higher AUC overall. LSA and Emb Cos both perform poorly, LSA performs only slightly better than random.

### Extracted-relationship based time-slicing results

**Fig. 5:**
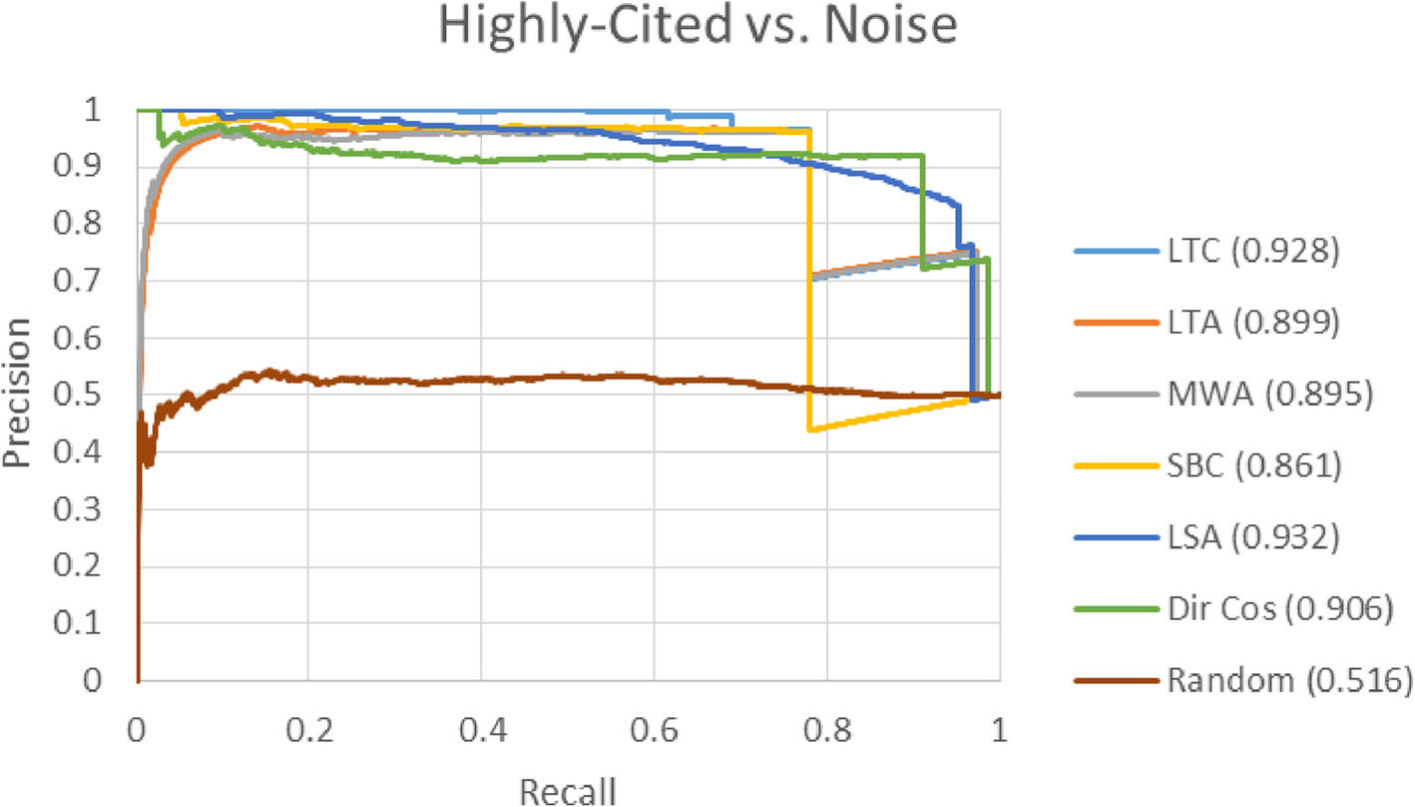
Highly-cited versus noise PR curve. This PR curve shows the ability of each ranking method to distinguish between *highly-cited* term pairs and *noise*. *Highly-cited* pairs appear in papers with over 100 citations after the cutoff date

**Fig. 6:**
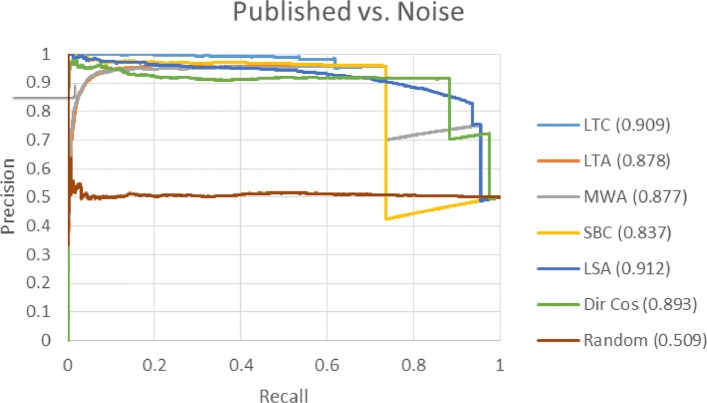
Published versus noise PR curve. This PR curve shows the ability of each ranking method to distinguish between *published* term pairs and *noise*. *Published* pairs appear in at least one paper after the cutoff date

In this section, we present the results using the extracted-relationship based time slicing datasets developed by Sybrandt et al. [6]. Figures 5 and 6 show the PR curves for the *highly-cited* versus *noise*, and *published* versus *noise* datasets respectively. Each colored line corresponds to a different target term ranking method. Figure legends show the area under the curve (AUC) in parentheses. Results were not generated for Emb Cos because it is unclear how to best generate concept embeddings from SemMedDB data.

For both the *highly-cited* versus *noise* and *published* versus *noise* datasets, results are very similar. The order of performance based on AUC from best to worst for both datasets is LSA, LTC, Dir Cos, LTA, MWA, and SBC. The vertical drops in performance seen in both figures are results of our tie-breaking rule. When term pairs have the same rank, false pairs are ranked higher, which results in vertical drops in precision until all tied terms have been ranked. LTA and MWA have low precision for low levels of recall indicating that the top ranked terms are *noise* term pairs. SBC and LSA have good precision for low levels of recall, so they may be preferred over LTA and MWA due to the importance of the top ranked terms. LTC has the highest precision for recall levels greater than 0.5, so it may be preferred over all other measures.

Sybrandt et al. [6] report the results of several ranking methods using ROC curves. To compare against their measures, we generated ROC curves (not shown) in a similar manner to PR curves, and calculated the area under the ROC (AUROC) curve for each of our evaluated methods. Table 5 shows the AUROC scores of our evaluated measures and their best performing measure, PolyMultiple. All our evaluated methods except SBC and random outperform PolyMultiple on both datasets.

**Table 5:** AUROC Scores for Comparison against Sybrandt

Our AUROC scores versus Sybrandt et al.		
Method	Highly-Cited vs. Noise	Published vs. Noise
Random	0.526	0.515
LTC	0.909	0.883
LTA	0.903	0.876
MWA	0.899	0.876
SBC	0.773	0.728
LSA	0.925	0.907
Dir Cos	0.919	0.906
PolyMultiple	0.874	0.834

The AUROC scores we generated using Sybrandt et al. code and the AUROC scores for their best performing methods

Upon analysis of Sybrandt et al. dataset, we found 7 and 13 term pairs from the *highly-cited* and *published* sets that exist in our pre-cutoff segment. The reason for this is unclear, since we both use SemMedDB for our pre-cutoff dataset, but it is possibly due to differences in SemMedDB versions. No term pairs from the noise dataset were present in our pre-cutoff segment.

## Discussion

**Fig. 7:**
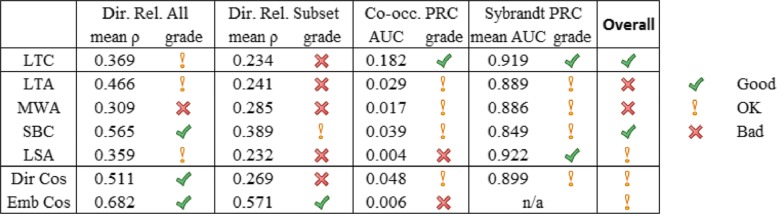
Performance comparison of evaluated methods. The average performance of each ranking method on the intrinsic task of estimating semantic relatedness, and the extrinsic task of ranking hypotheses in LBD. A grade of good, OK, or bad is assigned to each method to summarize the results

We evaluated four indirect association measures, LTA, MWA, SBC, and LSA on the extrinsic task of ranking hypotheses in LBD using PR curve analysis, and on the intrinsic task of estimating semantic similarity and relatedness. Results were also calculated for the baseline methods of LTC, direct cosine (Dir Cos) and embeddings cosine (Emb Cos). To better understand each evaluated measure’s overall performance, we summarized their results in Fig. 7. Each row shows the results of a different method and a “grade” of their performance. The grades were assigned in a somewhat subjective manner based on their order of performance and any noticeable groupings. The performance of measures varied depending on the evaluation methods and datasets, but this is understandable since each intrinsic and extrinsic evaluation method measures different aspects of performance, and the extrinsic time-slicing datasets are constructed in much different manners.

The first columns of Fig. 7 show performance for the intrinsic evaluation task of estimating semantic similarity and relatedness. For intrinsic evaluation, we measure the ability to estimate how similar or related two terms are, and use human-generated gold standard datasets of term pairs that directly co-occur. The “Dir. Rel. All” columns shows results for estimating direct relatedness using the full UMNSRS and Mini Mayo datasets. They show the mean Spearman’s rank correlation averaged across all four datasets, and a grade of their performance. Emb Cos, SBC, and Dir Cos all perform well. LTA, LTC, and LSA perform OK, and MWA performs poorly. The “Dir. Rel. Subset” columns shows results for estimating direct relatedness of the subsets of UMNSRS Sim and UMNSRS Rel using the *Disorders* and *Chemicals and Drugs* concept pairs. They show the mean Spearman’s rank correlation across both datasets, and a grade of their performance. All methods perform poorly, with the exceptions of SBC and Emb Cos, which perform OK and well respectively.

The next columns show performance for the extrinsic evaluation task of ranking target terms for LBD. For extrinsic evaluation, we measure the ability to rank target terms in LBD, and use automatically generated silver standard datasets of term pairs that do not co-occur. The “Co-occ PRC” columns show the results using our co-occurrence based time-slicing dataset. The AUC and a grade are shown. LTC performs the best. LTA, MWA, SBC, and Dir Cos performed similarly and performed OK, and LSA and Emb Cos performed the worst. The “Sybrandt PRC” columns shows the results using Sybrandt et al. [6]’s extracted-relationship based time-slicing dataset. All methods performed relatively well for this dataset, but LTC and LSA performed the best. All other methods performed OK. Results for Emb Cos were not generated for this dataset, since it is unclear how to best create concept embeddings using SemMedDB predication data.

Although LTC performs much better on our co-occurrence based extrinsic evaluation dataset, it performs similar to other methods on Sybrandt et al. extracted-relationship based extrinsic evaluation dataset, and worse than most methods for both intrinsic evaluation datasets. Co-occurrence based time-slicing datasets have been criticized as being too imprecise to effectively evaluate LBD. Since performance is similar for all methods using Sybrandt et al. dataset, which has higher precision, it’s possible that LTC’s good performance on our co-occurrence based dataset is a result of this low precision. Similarly though, Sybrandt et al. dataset has low recall, and other bias issues. This is why we used multiple evaluation datasets, and ideally a hypothesis ranking method should perform well for all datasets of both intrinsic and extrinsic evaluation tasks. LTC and SBC perform well, or OK on all evaluation datasets. SBC is one of the best performing methods for intrinsic evaluation, and has decent performance for both extrinsic evaluation datasets.

Emb Cos performs the best for intrinsic evaluation, but poorly for extrinsic evaluation, indicating that it is good at estimating relatedness between directly, but not indirectly co-occurring term pairs. LTA and MWA have consistently OK to bad performance across all datasets, indicating that although their ability to estimate relatedness extends to indirectly co-occurring terms, they don’t do a great job at it. LSA doesn’t perform well at estimating direct relatedness, and performs well on just a single extrinsic evaluation dataset. Its good performance on Sybrandt et al. datasets is due partially to its higher than average precision rates at high levels of recall, rather than having particularly high precision overall. Dir Cos appears to be one of the better performing measures, but on Sybrandt et al. dataset it never achieves very high levels of precision, and like LSA gets a higher AUC due to higher precisions at high levels of recall.

It is surprising that Emb Cos performs the best at estimating direct relatedness, but poorly for target term ranking in LBD, and that LTC performs the best for target term ranking, but just OK for the estimating direct relatedness. This shows the difference in estimating relatedness between directly versus indirectly co-occurring terms. It highlights differences in, and biases of the different evaluation techniques, and this along with differences in results for each extrinsic evaluation dataset highlights the need for a standard evaluation dataset that addresses the biases present in each of our evaluation datasets.

The difference in performance between SBC and LSA is surprising since their methodologies are similar, but indicates that their performance may be sensitive to the selection of the proxy sets for *A* and *C*. We believe LSA performs poorly because the *B*_*A*_ and *B*_*C*_ sets are too large and too noisy. SBC uses the shared linking term set, which is much smaller and more relevant to how *A* and *C* interact. Interestingly, direct cosine also uses the overlap of co-occurring terms or shared contexts, since only concepts that co-occur with *A* and *C* (and therefore are non-zero) contribute to the cosine distance. Filtering, or selecting only the most relevant terms for LSA, SBC, and direct cosine may improve results in the future.

Even though the performances of each method varied, having a variety of ranking methods with different theoretical foundations is useful; it allows the best method to be selected for each application. Preliminary collaborative efforts show that researchers often know the types of connections they are looking for, and want to fix both the *A* and *B* term sets to determine how some known *A* interacts with some *C* term set via the means of a relatively small, known *B* set (e.g. how a drug affects a class of diseases through means of several metabolites). In this scenario, it is likely that most terms will co-occur with the entire *B* term set, which means that each evaluated method except MWA would produce uninteresting results. LTC, LTA, and LSA require that the linking terms between *A* and *C* are different in order to produce interesting results. SBC and direct cosine produce similarly uninteresting results, since restricting the *B* set restricts the shared *B* set making results identical for all *A* and *C* terms. Emb Cos takes the cosine between *A* and *C* vectors and ignores the *B* terms entirely, and therefore how *A* interacts with *C* through *B*. So, although MWA performs poorly at estimating future relatedness, MWA is the only method capable of producing interesting rankings in this scenario. To further differentiate each evaluated method, we divide the methods into three groups, linking term based methods (LTC, LTA, MWA), which directly use the linking terms in their calculations. Set based association methods (SBC, LSA), which use set associations between proxy sets in their calculations. Vector methods (Dir Cos, Emb Cos) which use vector cosine to quantify relatedness. Below, we summarize the differences between evaluated measures and indicate their strengths:

- LTC - linking term based, which is simple to compute and has the best empirical performance for link prediction.
- LTA - linking term based method, is faster to compute than other indirect association measures.
- MWA - linking term based method, which may be the only interesting method when *B* terms are restricted to a small set.
- SBC - set based association method, which performs well at all tasks making this a good general purpose indirect association measure.
- LSA - set based association method, which performs poorly at most tasks, but uses the largest sets of proxy terms. This gives it the greatest chance of being able to quantify relatedness, and could therefore be useful in domains with small datasets
- Dir Cos - vector method, which performs well on all tasks (except Dir. Rel. Subset), making this a decent, simple to compute, general purpose method.
- Emb Cos - vector method, good for estimating direct relatedness. This is the only method that does not rely on a co-occurrence matrix, which makes it the fastest to compute.

## Conclusions

In conclusion, we evaluated four indirect association measures, LTA, MWA, SBC, and LSA against and baselines of LTC, direct co-occurrence vector cosine, and concept embeddings cosine for the intrinsic evaluation task of estimating semantic similarity and relatedness, and the extrinsic evaluation task of ranking hypotheses in LBD. We used a gold standard, human graded dataset for intrinsic evaluation, but it only evaluates performance using directly co-occurring terms. To evaluate for terms that never directly co-occur, we used two different extrinsic evaluation datasets, a co-occurrence based time-slicing dataset, and an extracted-relationship based time-slicing dataset. These silver standard time-slicing datasets both imperfectly estimate the gold standard of all possible future discoveries, but have different characteristics and biases. The co-occurrence based dataset has high recall, but low precision, and the extracted-relationship based dataset has low recall, but higher precision. Results differed based on the evaluation method and dataset, but overall we found that LTC and SBC are the best performing methods for hypothesis ranking in LBD. This conclusion is based on SBC’s overall good performance, and LTC’s good performance on both extrinsic evaluation datasets.

## Abbreviations

AMW: Average minimum weight
AUC: Area under the curve
AUROC: Area under the receiver operating characteristic curve
CBOW: Continuous bag of words
Dir Cos: Direct co-occurrence vector cosine distance
Emb Cos: Concept embedding cosine distance
HAL: Hyperspace analogue to language
ICC: Intraclass correlation coefficient
LBD: Literature based discovery
LSA: Linking set association
LTA: Linking term association
LTC: Linking term count
MiniMayo Cod: The MiniMayoSRS dataset rated by medical coders
MiniMayo Phys: The MiniMayoSRS rated by physicians
MiniMayoSRS: Subset of MayoSRS reference standard
MWA: Minimum weight association
PR: Precision and recall
PSI: Predication-based semantic indexing
ROC: Receiver operating characteristic
SBC: Shared B to C set association
SNOMED CT: Systematized nomenclature of medicine: clinical terms
UMLS: United medical language system
UMNSRS: University of minnesota medical residents similarity/relatedness reference standard
UMNSRS Rel: The UMNSRS tagged for relatedness
UMNSRS Sim: The UMNSRS tagged for similarity
*ρ*: Spearman’s rank correlation coefficient

## Footnotes

1
https://metacpan.org/pod/UMLS::Association

2
http://www.people.vcu.edu/~henryst/indirectRanking.tar.gz

3
https://ii.nlm.nih.gov/MMBaseline/index.shtml

4
https://metacpan.org/pod/UMLS::Association

5
https://metacpan.org/pod/UMLS::Association

6
https://metacpan.org/pod/Word2vec::Word2vec
